# Case report: chronic acalculous cholecystitis preceded by Coxsackievirus B4 infection

**DOI:** 10.1093/jscr/rjac074

**Published:** 2022-03-15

**Authors:** Connor E Fewel, Joshua R Weiss, Jane C Harrington

**Affiliations:** Department of Microbiology, Immunology and Pharmacology School of Medicine, St. George's University, Grenada, West Indies; Department of Microbiology, Immunology and Pharmacology School of Medicine, St. George's University, Grenada, West Indies; Department of Microbiology, Immunology and Pharmacology School of Medicine, St. George's University, Grenada, West Indies

## Abstract

A 41-year-old female presented with an 8-month history of right upper quadrant pain, exacerbated by ingestion of saturated fats. The patient was positive for antibodies to Coxsackievirus serotype B4, established by an investigation incited by an acute episode of pleurodynia 8 months before the current presentation. Imaging studies including a hepatobiliary iminodiacetic acid scan showed no gallbladder structural or functional abnormalities. Laboratory studies indicated pancreatic enzyme insufficiency associated with below-normal lipase and amylase levels. Patient symptomology was consistent with cholecystitis with positive Murphy’s sign, so cholecystectomy was recommended. Post-surgery pathological report confirmed chronic acalculous cholecystitis. Patient demonstrated full recovery, indicated by return of normal pancreatic enzymes levels and resolution of abdominal pain.

## INTRODUCTION

Chronic acalculous cholecystitis (CAC) is an atypical presentation of gall bladder inflammation that is more challenging to diagnose clinically, compared with acute calculous cholecystitis. Negative imaging and lab studies associated with CAC patients can lead to missed diagnoses, thus potentially leaving patients with longer duration of restrictive right upper quadrant (RUQ) pain [1, 2]. Viral infections, including SARS-CoV2, have been associated with the onset of CAC; however, Enteroviruses, including coxsackievirus, have not previously been reported as a known trigger for gall bladder pathology. Coxsackievirus serotype B4 (CVB4) is a known cause of pancreatitis and pleurodynia (or Bornholm disease), which can present with pleuritic chest pain, hypochondrial pain, fever and pain in the muscles of the trunk/extremities [3]. This case report highlights a preceding infection CVB4 as a novel risk factor for the onset of CAC.

## CASE REPORT

**Figure 1:**
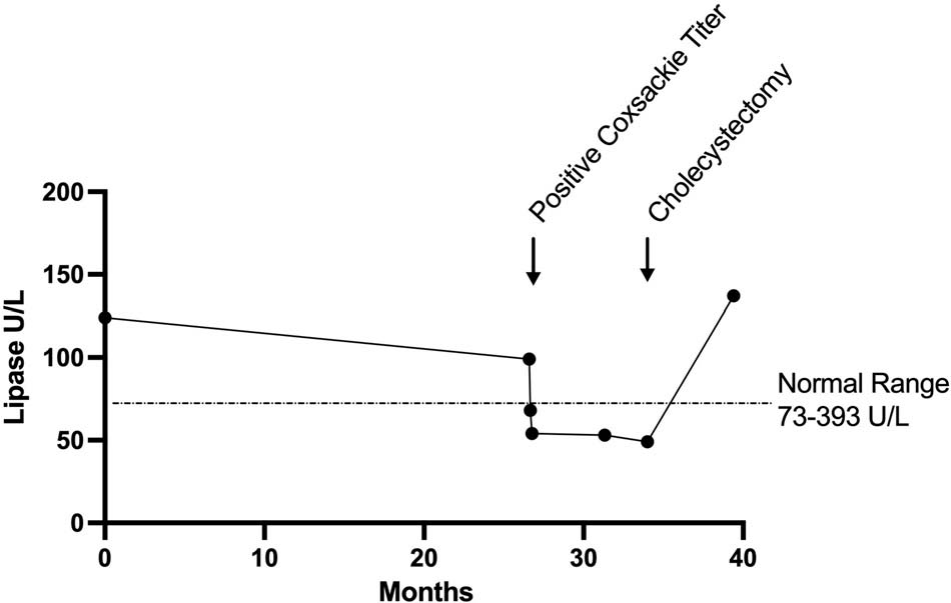
Decreased lipase levels associated with CVB4 infection. Serum samples were assessed for lipase levels to investigate complaints of abdominal pain. Lipase levels were within normal limits (73–393 U/L) at the time of GERD diagnosis, 4 years before the onset of RUQ pain. Levels were notably reduced below normal range, concurrent with episode of acute pleurodynia and remained low before the cholecystectomy.

A 41-year-old Caucasian female, G1 P1, presented to a gastroenterology clinic with RUQ pain and 45-lbs unintentional weight loss of 8-month duration. Medical history revealed that the patient was diagnosed with irritable bowel syndrome at age 17 and gastroesophageal reflux disease at age 37. An acute episode of left upper quadrant (LUQ) abdominal and lower chest pain preceded the onset of the RUQ pain. Imaging studies with CT scan and abdominal ultrasound conducted to investigate LUQ and chest pains showed focal inflammation in jejunum and bilateral chest atelectasis; however, no indication of gall bladder abnormality was reported. Laboratory investigations had demonstrated positive titer for CVB4 IgG value 1: 160 (ref < 1:10), below normal levels of amylase level (22 U/L, ref 30–110 U/L) and lipase (54 U/L, ref 73–373 U/L; Fig. 1). Patient was administered abdominal lidocaine shots, which mitigated the severity of the LUQ pain.

The patient reported that the upper abdominal pain progressed from LUQ to RUQ after the pleurodynia episode. The pain was described as constant throbbing and was exacerbated by the consumption of saturated fats consistent, with biliary pathology [2]. Her vital signs remained within normal limits. Imaging with RUQ ultrasound and hepatobiliary iminodiacetic acid with cholecystokinin CCK injection showed normal gall bladder structure and function (53% ejection rate). Despite normal imaging, cholecystectomy was recommended based on the characteristic of cholecystitis symptomology, including a positive Murphy’s sign. A positive Murphy’s sign has a 97% sensitivity and 46% specificity for predicting cholecystitis [2]. Pre-surgery laboratory bloodwork indicated that the lipase level had remained below normal (49 U/L, ref 73–373 U/L; Fig. 1). Gallbladder pathology report post-surgery confirmed CAC. All abdominal pain symptoms abated post-surgery and lipase levels returned to within normal limits (Fig. 1).

## DISCUSSION

CAC has historically been a diagnosis of exclusion following negative imaging studies with positive symptomatology [1]. The diagnostic criteria of CAC are similar to acute cholecystitis caused by cholelithiasis highlighted by mononuclear infiltrate, fibrosis and metaplasia on post-cholecystectomy pathology [4]. Given the elusive nature of diagnosing CAC preoperatively, the prevalence of disease is likely under reported. Acalculous cholecystitis has been estimated to account for between 50 and 70% of cholecystitis in children with the most common precipitating events being dehydration, bacterial infection, viral infection (e.g. herpes simplex virus, hepatitis A virus) and upper respiratory tract infection [5]. More recent studies suggest that the pathogenic mechanism may be very similar in adult patients [6].

Case reports of viral infections affecting either the liver or pancreas have been established to induce acalculous cholecystitis in both pediatric and adult populations. Notable causes of viral cholecystitis, include SARS-Cov-2 [7, 8], Hepatitis A [9] and Epstein–Barr Virus [10]. Coxsackieviruses, members of the *Picornaviridae* Family, Enterovirus Genus, have not been attributed to onset of gall bladder disease, before this case report. Coxsackievirus Group B (CVB4) is a well-established causative agent for pancreatitis and pleurodynia, ranging from acute to chronic disease [11]. The hallmark indicator of viral-induced acute pancreatitis is lipase levels above normal range; however, the lipase levels of the patient remained below normal range after the onset of pleurodynia (Fig. 1). The coxsackievirus-adenovirus receptor and the decay-accelerating factor are the notable receptor proteins that play an important role in the pathogenesis of CVB4 infections [3]. The vagus nerve and the biliary tract both provide direct routes between common Coxsackievirus infection locations in the gastrointestinal lining and pancreas to the abdominal and chest pleura. The gallbladder endothelium may be a potential reservoir for CVB4 infection due to the viral broad tropism affinity. The direct anatomical connections, in conjunction with prior reports of cholecystitis stemming abnormally from neighboring virally infected organs, lead to a speculation that the prior CVB4 infection in the patient contributed to the onset of CAC.

The concomitant findings of a positive CVB4 IgG titer and persistently low levels of pancreatic enzymes provide stronger evidence of the infection timeline. Enteroviruses, including CVB4, have been established to cause transient exocrine pancreatic insufficiency for longer than 1.5 months [12]. In this patient, the first laboratory evidence of pancreatic insufficiency was documented at the onset of pleurodynia, providing strong evidence of active CVB4 (Fig. 1). The patient’s exocrine pancreatic insufficiency persisted until there was spontaneous resolution post-cholecystectomy.

This case study highlights a previously undocumented risk factor of Coxsackievirus infection for the development of CAC. Clinicians can consider the combinatory factors in conjunction with physical examination findings, laboratory findings and symptomology to recommend treatment in patients who yield negative imaging studies. Furthermore, identifying new risk factors of CAC will contribute a better understanding of an enigmatic disease, thus allowing clinicians to advise specific patient populations to modify their lifestyle to prevent the development of complications.

## STATEMENT OF ETHICS

### Ethical Review

The investigations of this case report were compliant with ethical practices established by Nuremburg Code. A written informed consent was submitted by the subject patient. The case study was submitted by the Internal Review Board at St. George’s University and was determined to fit the criteria for exemption for full review.

## AUTHOR CONTRIBUTIONS

The authors of the case study contributed equally to the development, analysis and writing of the case study.

## CONFLICT OF INTEREST STATEMENT

The authors of the case study have no conflicting established partnerships of financial or non-financial support. The authors of the study hold academic roles (faculty and students) at St. George’s University, West Indies and have no non-academic affiliations. The senior author is the subject patient and has no external conflicts of interest.
